# Intrarenal arteriovenous malformation following flexible ureterorenoscopy and holmium laser stone fragmentation: report of a case

**DOI:** 10.1186/s12894-019-0447-7

**Published:** 2019-03-22

**Authors:** Abdelrahman Bashar, Fayez T. Hammad

**Affiliations:** 1 Urology Division, Tawam Hospital in affiliation with Johns Hopkins Medicine, Al Ain, UAE; 2 Department of Surgery, College of Medicine and Health Sciences, United Arab Emirates University, Po Box 17666, Al Ain, UAE

**Keywords:** Arteriovenous malformation, Flexible ureterorenoscopy, Holmium laser

## Abstract

**Background:**

Flexible ureterorenoscopy (FURS) and holmium laser lithotripsy is currently considered as one of the treatment options for large renal calculi. It has been shown to be safer than percutaneous nephrolithotomy. The latter can sometimes be complicated by the formation of intrarenal arteriovenous malformation (AVM). AVM is extremely rare following FURS and laser lithotripsy. Indeed only one case has been reported on reviewing the literature up to June 2018. We report on the second case illustrating the possibility of developing this major complication following this procedure.

**Case presentation:**

A 79 years old diabetic and hypertensive male with stage-4 chronic kidney disease who previously had left extracorporeal shockwave lithotripsy and FURS with Holmium laser lithotripsy, presented with bilateral large renal calculi. He underwent simultaneous bilateral FURS and Holmium laser lithotripsy and was discharged home the following day with almost clear urine. Four days post-discharge, he presented with gross hematuria for which he required hospitalization and blood transfusion. CT scan demonstrated left subcapsular, perinephric and retroperitoneal hematoma. Angiography showed contrast extravasation from pseudoaneurysms in two small branches of left renal artery. Both were selectively embolized with micro-coils and this led to the cessation of the hematuria.

**Conclusions:**

Despite the relative safety of FURS and Holmium laser lithotripsy, it can be associated with major complications like intrarenal AVM. This can probably be prevented by careful and judicious use of laser energy in patients with large stone burden and premorbid conditions.

## Background

The recent and on-going advances in flexible instrumentation and laser technology have expanded the indications of retrograde intrarenal surgery. As per many guidelines, flexible ureterorenoscopy (FURS) and holmium laser lithotripsy is currently considered as one of the treatment options for renal stones larger than 2 cm in diameter. It has less complication rate compared to the more invasive procedures such as percutaneous nephrolithotomy [1, 2]. Following percutaneous nephrolithotomy, the formation of intrarenal arteriovenous malformations (AVM) in the form of arteriovenous fistulae or pseudoaneurysms, although rare with an incidence of around 1.4%, is a well-known complication [3, 4]. However, this complication is extremely rare following FURS and holmium laser lithotripsy. To our knowledge, and by reviewing the English literature in the Medline up to June 2018, there was only one case of reported AVM following this procedure [5]. Herein, we report on the second case of AVM following FURS and holmium laser lithotripsy, which presented with delayed hematuria and required angio-embolisation.

## Case report

**Fig. 1:**
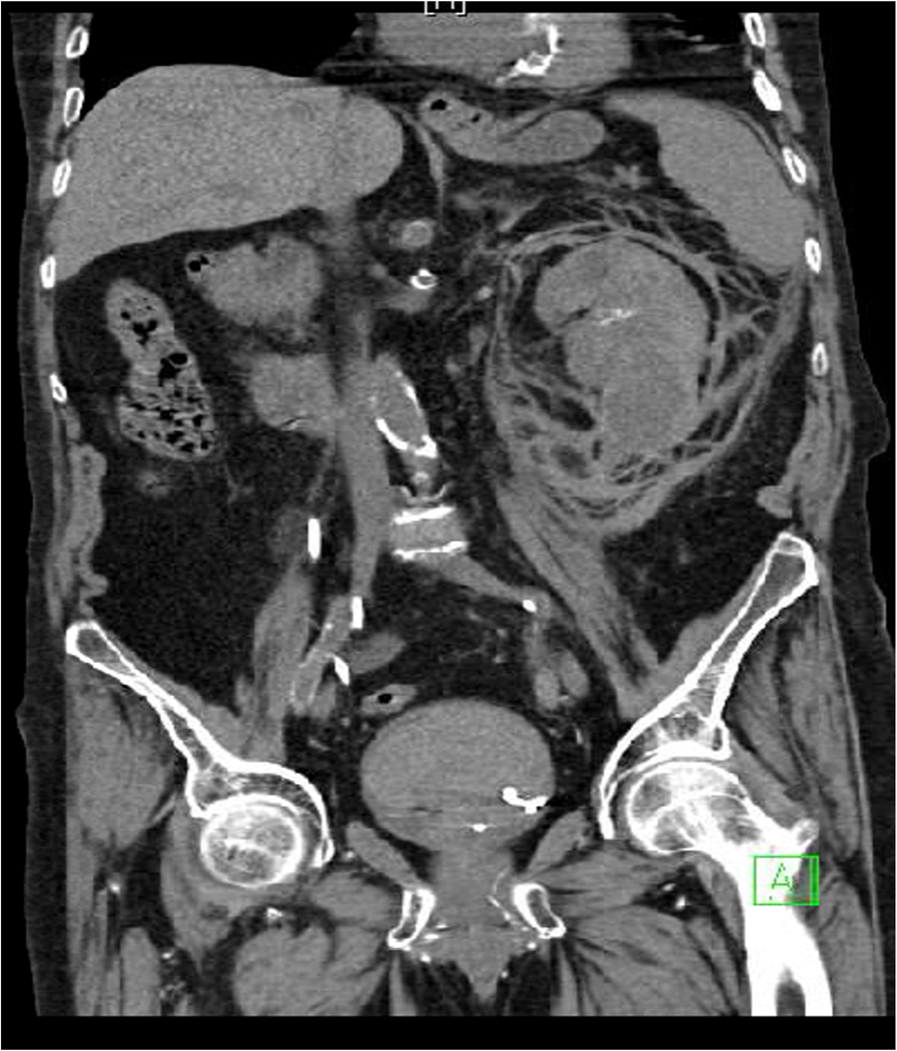
Coronal view of the non-contrast CT scan showing severe left-sided perinephric and retroperitoneal hematoma. It also demonstrates the intra-vesical part of the dislodged double coil stent. Minimal stone fragments are left over

**Fig. 2:**
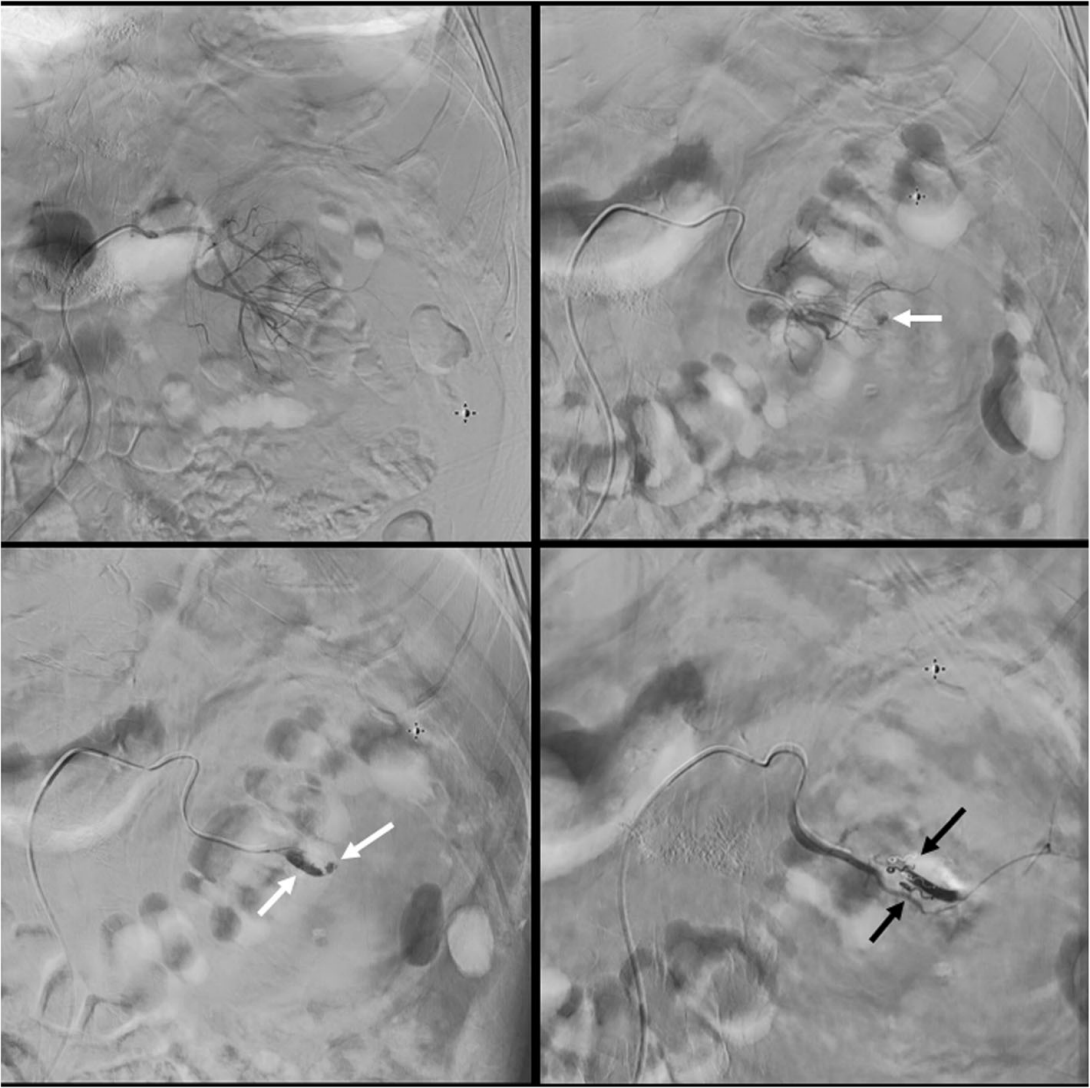
Lower pole selective angiogram showing two small pseudo aneurysms with extravasation of contrast material outside the renal parenchyma (light arrows). Selective embolisation of both branches using microcoils (dark arrows)

A 79 years old male with multiple comorbidities including hypertension, valvular heart disease, diabetes mellitus and stage 4 chronic kidney disease with a baseline creatinine of more than 300 μmol/L presented with bilateral symptomatic large renal stones for which he underwent staged stone treatment. Prior to presentation to our hospital, he had bilateral double J stent (DJS) insertion and left extracorporeal shockwave lithotripsy followed by FURS and laser stone fragmentation of the left renal stones. Subsequently, he sought medical advice in our facility. Non-contrast CT scan showed multiple bilateral renal stones. In the left kidney, there were 3 stones distributed to middle and lower pole calyces with a stone burden of approximately 3.0 cm as measured using the CT scan. In the right kidney there were also three stones, two in middle calyces and one in the pelvis with a total stone burden of 3.2 cm. After stopping the aspirin for seven days, he underwent simultaneous bilateral FURS and holmium laser lithotripsy and insertion of bilateral DJS under general anesthesia with endotracheal intubation. The surgical procedure took 125 min (65 min for the left side followed by 60 min for the right one) and the procedure was similar in both sides. Following insertion of a hydrophilic tip guidewire (Sensor, 0.038 in), a ureteral access sheath (Inner diameter: 12 Fr, Length: 55 cm) was inserted and the tip was located approximated at the level of ureteropelvic junction. Karl Storz flexible ureterorenoscope (8.5 Fr) was used. During the procedure the normal saline was allowed to run from the bag (approximately 80 cm above the level of the patient pelvis without a pump) and the outflow of saline from around the scope was observed throughout the procedure. Laser energy between 1.0–1.2 joules with a frequency ranging between 8 and 12 Hz (short pulses) were used in both sides. 4200 and 4066 pulses were used in the left and right sides respectively. He was discharged home in a good condition the next day with almost clear urine. Four days later, he reported back to the emergency department with severe suprapubic pain and gross hematuria which required continuous bladder irrigation with a three-way catheter. His hemoglobin dropped dramatically from 121 to 84 g/dl requiring an initial transfusion of two units of packed red blood cells. CT scan showed left-sided subcapsular, perinephric and retroperitoneal hematoma (Fig. 1), bladder clots and complete dislodgement of the left DJS. Cystoscopy revealed bleeding from the left ureteric orifice. Evacuation of clots and change of the DJS were performed. Postoperatively, he continued to have hematuria which was initially treated conservatively with bladder irrigation. His Hb dropped again from 113 to 85 g/dl and in view of the ongoing hematuria he required an additional five units of packed red blood cells. In view of the impaired renal functions, the patient was initially reluctant to have angiogram but ultimately it was performed and showed pseudoaneurysms in two small branches of the left main renal artery with extravasation of the contrast outside the renal parenchyma (Fig. 2). Both branches were then selectively embolized using 3x2mm microcoils. As results, the hematuria ceased and he was discharged home two days later. On follow-up, his creatinine remained similar to the pre-operative values and in view of the patient comorbidities and of what happened during this procedure; he decided not to go for another procedures for the residual stones.

## Discussion and conclusions

Like all other types of surgery, the use of FURS and holmium laser lithotripsy is not without complications. These complications, although relatively rare, include urosepsis, mucosal injury and bleeding [6, 7]. Unlike percutaneous renal stone surgery [3, 4], the formation of AVM malformation is extremely rare following FURS and holmium laser lithotripsy. To our knowledge, only one case of AVM was reported following this procedure [5]. Our case is another evidence of the possible occurrence of AVM following the use of FURS and holmium laser lithotripsy.

The exact etiology for the formation of AVM in our case is difficult to ascertain and could be multifactorial. Theoretically, the AVM could have formed as a result of the possible mechanical trauma to the renal tissues by the laser probe or guidewire during the initial manipulation. The fact that there was no significant bleeding soon after the procedure and the fact that the hematuria was delayed till the fourth post-operative day refute this possibility. A more likely possibility would be the thermal injury caused by laser lithotripsy during stone fragmentation as suggested by Tiplitsky et al. [5]. Additional contributing factors in our case could be the previous treatment with FURS and laser stone fragmentation and extracorporeal shock wave lithotripsy which could have affected the renal vessels making them more liable for the laser-induced trauma [8, 9]. Further, and despite the observation of free outflow of saline from the access sheath around the scope during the procedure a high intrarenal pressure could have been reached leading to renal dilation and further thinning of the parenchyma during the procedure and hence exacerbated the injury of blood vessels. Moreover, the cardiovascular disease and renal impairment and the associated effect on renal vasculature in this patient could have also exacerbated the laser effect [10]. Collectively, it is more likely that the formation of AVM was due to the use of laser lithotripsy rather than the use of FURS per se. With this regard, other lithotripsy modalities such as electrohydraulic lithotripsy was also shown to result in intra-renal AVM although extremely rare and indeed only one case was reported following the use of FURS and electrohydraulic lithotripsy [10].

Regardless of the exact cause of the AVM post-FURS and laser lithotripsy, a high index of suspension is required to diagnose these cases when such patients present with delayed hematuria. Moreover, every effort should be made to decrease the laser-induced trauma to renal tissues. This could be achieved by careful use of guidewires and laser probes and keeping them away from the urothelium as much as possible and by using the minimum energy required to fragment the stone especially in patients with underlying morbidities which affect the integrity of renal vasculature. Every effort should be made to insure a clear vision and low intrarenal pressure and certainly multi-stage procedures should be performed instead of one long-procedure especially when there is poor visibility and large stones. Further, despite the rarity of this complication, it might need to be discussed during pre-operative counselling of high risk patients.

## Abbreviations

AVM: arteriovenous malformation
DJS: double J stent
FURS: flexible ureterorenoscopy
